# Tumoral Calcinosis of the Thoracolumbar Spine Associated With Adjacent Segment Degeneration After Lumber Fusion: A Case of Myelopathy

**DOI:** 10.7759/cureus.79851

**Published:** 2025-02-28

**Authors:** Yu Kobayashi, Takaki Inoue, Hiroyuki Motegi

**Affiliations:** 1 Department of Orthopaedic Surgery, Chiba Aoba Municipal Hospital, Chiba, JPN

**Keywords:** adjacent segment degeneration, myelopathy, posterior decompression and fixation, thoracolumbar spine, tumoral calcinosis

## Abstract

Tumoral calcinosis is characterized by the deposition of calcified masses in peri-articular tissues, typically near joints. Spinal involvement is rare, particularly following adjacent segment degeneration (ASD) after lumbar spinal fusion. We present the case of a 73-year-old female who developed tumoral calcinosis with myelopathy following lumbar fusion surgery. She had previously undergone two lumbar spine surgeries, which resulted in lumbar fusion from L2 to L5. She developed lower back and leg pain, which progressively worsened, eventually leading to bilateral lower limb paralysis, paresthesia, bladder and rectal dysfunction, and gait disturbance. Computed tomography revealed calcified lesions at the T12-L1 segment adjacent to the spinal fusion. Surgical treatment included laminectomy with resection of the calcified lesions for decompression and extended fusion for stabilization. Complete removal of the calcified lesions via a posterior approach was challenging due to its extensive anterior involvement; therefore, partial resection was performed. A white calcified substance was extracted from the lesion, and ultrasound confirmed adequate decompression of the spinal cord. Postoperatively, the patient showed significant neurological improvement, and follow-up imaging showed no progression of the calcified lesion. This case highlights the importance of considering tumoral calcinosis in the differential diagnosis of spinal cord compression following ASD after spinal fusion. Spinal instability due to ASD may contribute to the development of spinal tumoral calcinosis, and surgical decompression and stabilization appear to be effective treatment options.

## Introduction

Tumoral calcinosis is a pathologic entity characterized by radiodense peri-articular masses caused by dystrophic calcification in soft tissue. It is typically found near large joints, with the most common locations, in descending order, being the hip, elbow, shoulder, foot, and wrist [1]. Tumoral calcinosis involving the spine is rare [2]. ﻿This condition is a benign hereditary disorder that occurs primarily in young adults and often exhibits familial clustering. It may also be associated with underlying conditions such as chronic renal failure, hyperparathyroidism, hypervitaminosis D, scleroderma, pseudoxanthoma elasticum, malignancy, and milk-alkali syndrome [3]. In addition, vertebral disc and facet joint degeneration have been identified as potential contributing factors in the development of spinal tumoral calcinosis [2,4,5]. Diagnosis is based on clinical evaluation and imaging modalities, including radiography, computed tomography (CT), magnetic resonance imaging (MRI), and histopathological analysis [5,6]. The lesions typically grow slowly and remain asymptomatic; however, they can cause pain and neurological symptoms when there is nerve involvement. In cases where spinal tumoral calcinosis leads to progressive neurological deficits, surgical removal is the treatment of choice [7]. To date, no cases of adjacent segment degeneration (ASD) following lumbar fusion surgery leading to tumoral calcinosis have been reported. Here, we present a case of tumoral calcinosis accompanied by myelopathy due to ASD after lumbar spinal fusion.

## Case presentation

**Figure 1:**
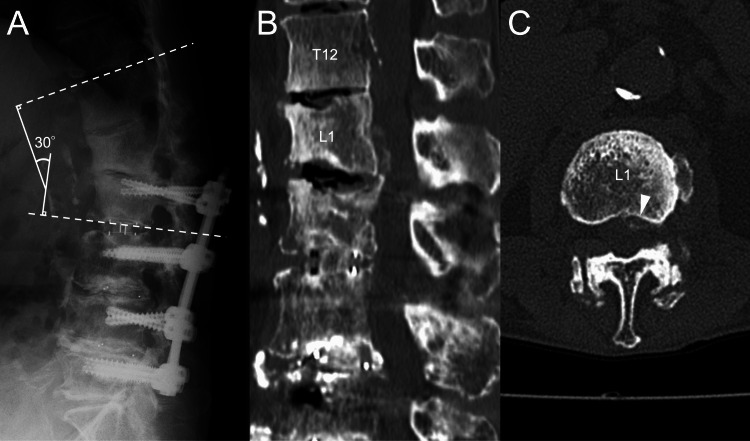
Image taken a year ago. (A) Lateral neutral radiographs show the disappearance of the disc space at L1-L2 and a kyphotic deformity (T12-L2 Cobb angle = 30°). (B) Sagittal CT image. (C) Axial CT image reveals a pale calcified lesion (indicated by white arrowhead) in the ventral epidural space at the L1 level.

**Figure 2:**
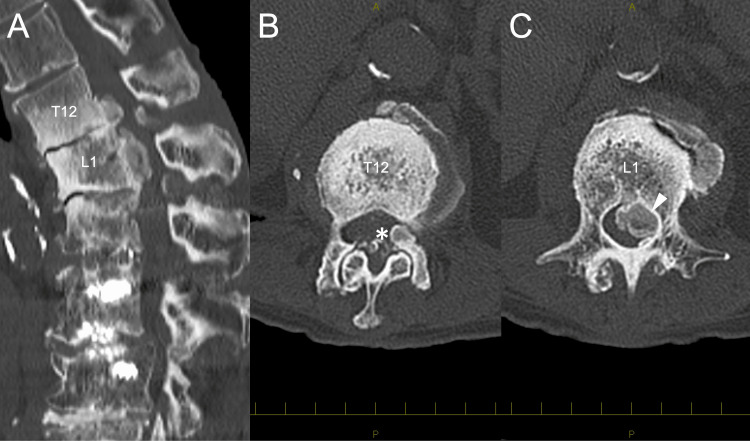
Preoperative CT images (A) Sagittal CT scan reveals the enlarged calcified lesion in ventral epidural space at T12-L1. (B) Axial CT image showsa calcified lesion (indicated by asterisk) at the anterolateral aspect of the left T12-L1 facet joint. (C) Axial CT imageshows the calcified lesion (indicated by white arrowhead) in the ventral epidural space at the L1 level.

A 73-year-old female with a history of duodenal ulcer had undergone two prior lumbar spine surgeries: transforaminal interbody fusion (TLIF) from L3 to L5 nine years ago for lumbar spinal canal stenosis and an extension of the spinal fusion to L2 with combined anterior and posterior approaches for ASD six years ago. These surgeries resulted in lumbar fusion from L2 to L5. One year ago, she began experiencing lower back and leg pain. Radiographs showed the disappearance of the disc space at L1-2 and the progression of local kyphosis (Figure 1A). CT revealed a pale calcified lesion in the ventral epidural space at the L1 level (Figures 1B-1C). Her clinician opted for conservative management with a rigid brace. However, three months ago, her symptoms progressed, leading to bilateral lower limb paralysis (1-3 according to the Manual Muscle Test), paresthesia, bladder-rectum disorder, and gait disturbance. Blood tests showed mild renal dysfunction (blood urea nitrogen: 10.7 mg/dL; creatinine: 0.64 mg/dL; eGFR: 68.2 ml/min/1.73 m²), but serum calcium (8.9 mEq/L), phosphorus (4.4 mEq/L), and parathyroid hormone levels were within normal ranges. CT revealed enlarged calcified lesions at the anterolateral aspect of the left T12-L1 facet joint and in the ventral epidural space at T12-L1 (Figure 2A-2C). 

**Figure 3:**
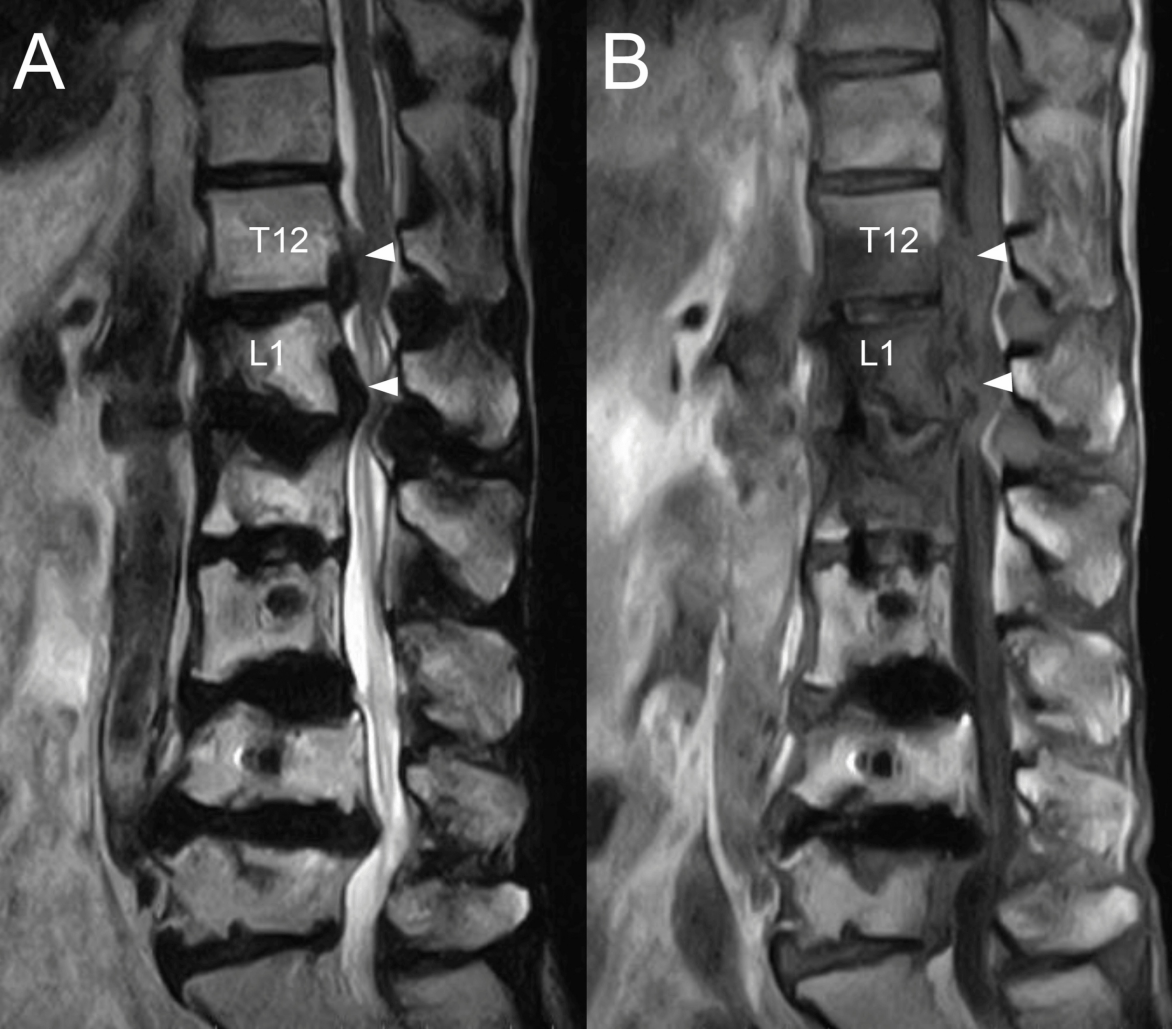
Preoperative MRI images Preoperative MRI images show the epidural lesion (indicated by white arrowhead), which appeared hypointense on T2-weighted images (A) and hypointense to isointense on T1-weighted images (B).

**Figure 4:**
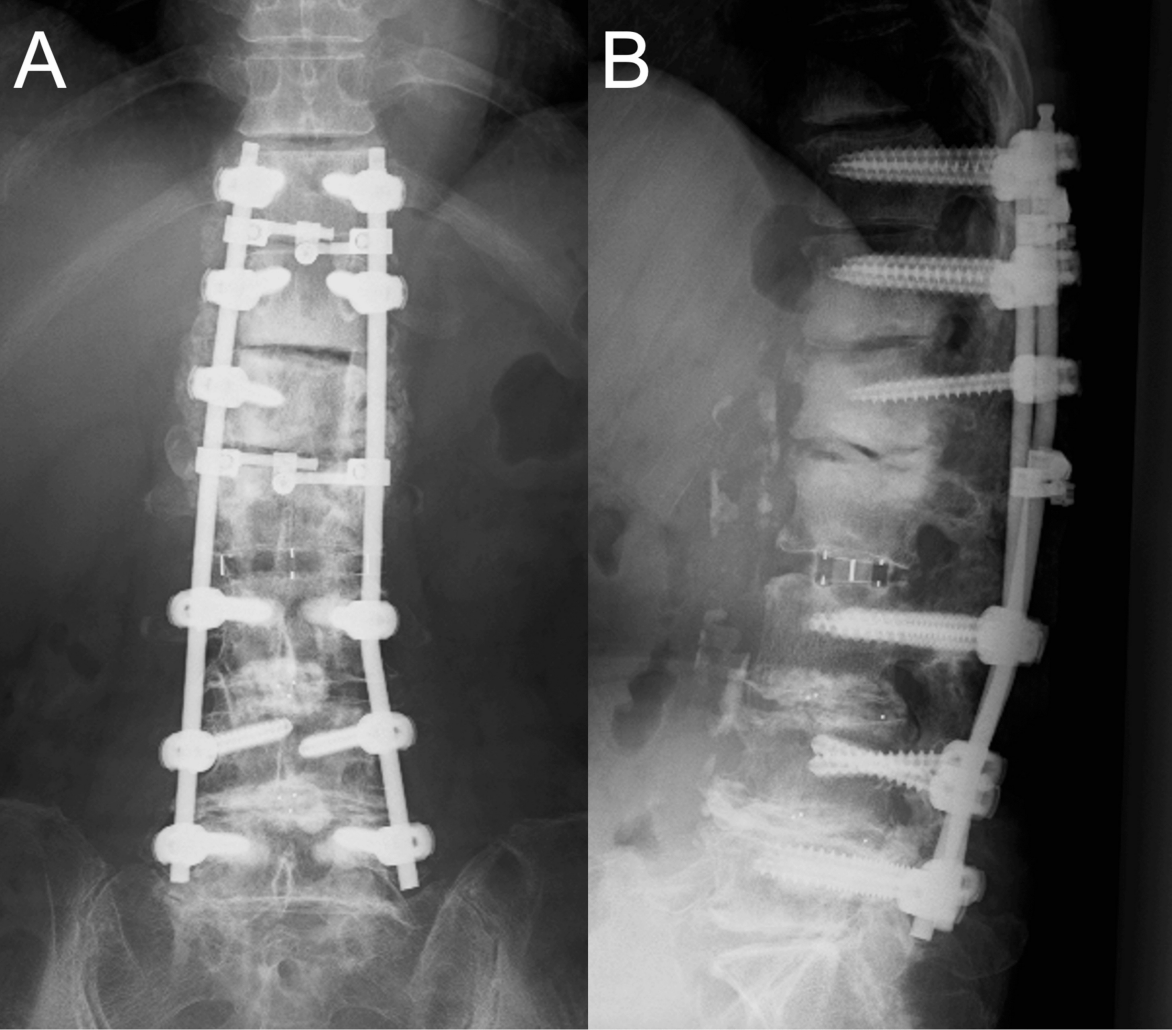
Postoperative radiography images. (A) Anteroposterior view. (B) Lateral neutral view.

**Figure 5:**
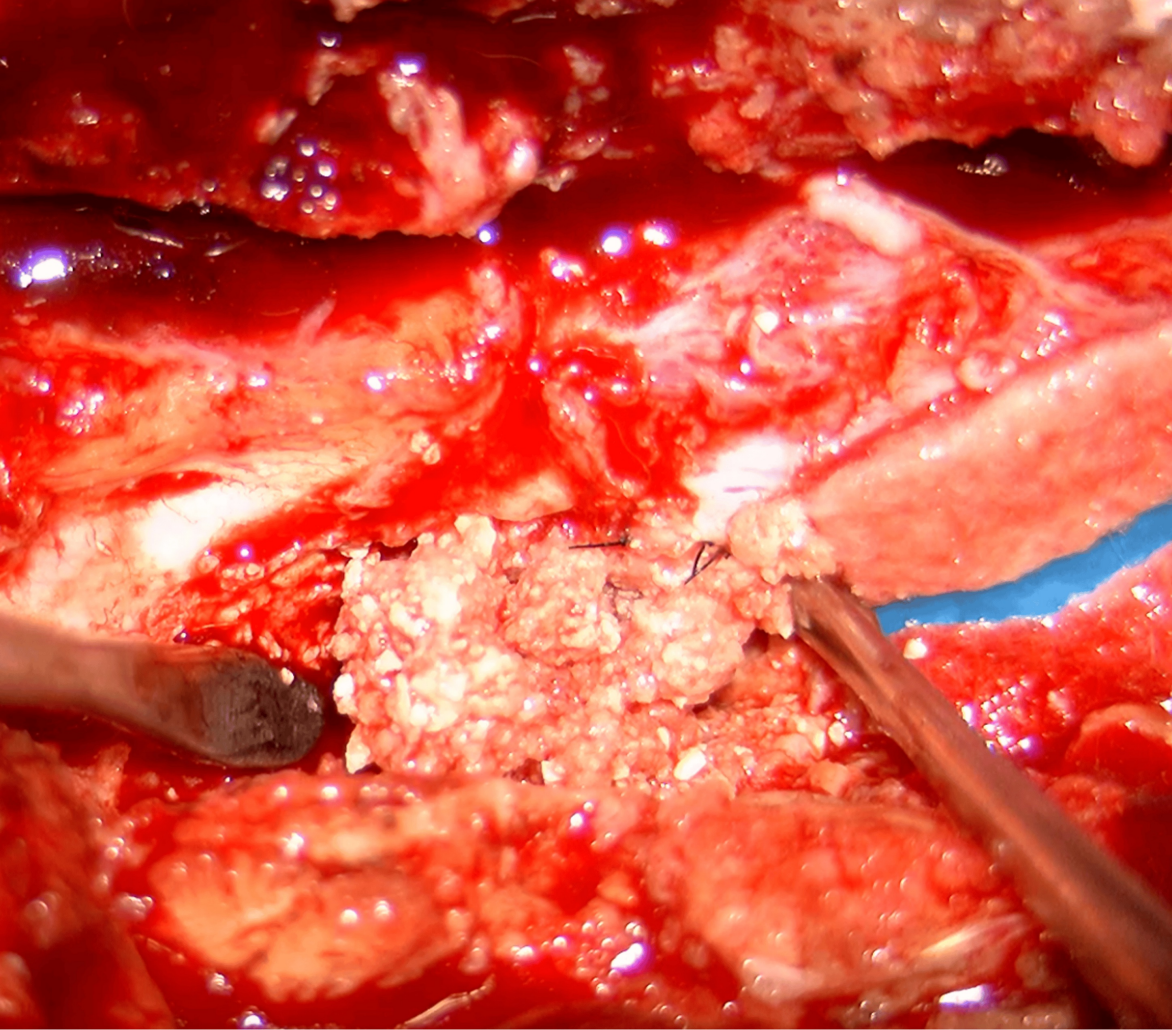
Intraoperative photograph A white calcified substance was extracted from the lesion intraoperatively.

MRI showed the epidural lesion that appeared hypointense on T2-weighted images and hypointense to isointense on T1-weighted images (Figure 3A-3B). The patient underwent decompression and extended fusion surgery (Figure 4A-4B). For decompression, a T12-L2 laminectomy was performed. The calcified lesion at the anterolateral aspect of the left T12-L1 facet joint was excised. To access the ventral epidural lesion via a posterior approach, the medial half of the T12-L1 facet joint and part of the L1 pedicle on the left side were resected. Complete resection of the ventral epidural lesion was challenging due to the risk of intraoperative neurological complications. Therefore, partial resection was carried out instead. A white calcified substance was extracted from the lesion, with no obvious pus (Figure 5). Ultrasound confirmed adequate decompression of the spinal cord. For extended fusion, the previously inserted bilateral L2 pedicle screws and rods were removed, and pedicle screws were inserted bilaterally at T11 and T12 and on the right side at L1, with rods threaded through the screw heads spanning from T11 to L5. In addition, bone grafts harvested from the iliac crest were placed on the bilateral decorticated transverse processes from T11 to L2 and the right T12-L1 and L1-2 facet joints. Pathological examination revealed degenerated ligamentous and necrotic tissue with associated dystrophic calcification. The crystals were not composed of calcium pyrophosphate or uric acid. Soft tissue cultures were negative. Postoperatively, her lower limb strength improved, and within three months, she was able to walk indoors with a cane. At the six-month follow-up, her neurological examination remained stable, and the residual calcified lesions showed regression. 

## Discussion

Tumoral calcinosis, which may occur following ASD after lumbar spinal fusion, should be considered in the differential diagnosis of spinal cord compression. Even when total excision of tumoral calcinosis is challenging, laminectomy with partial lesion resection and extended fusion may be effective in treating this pathological condition. 

Tumoral calcinosis typically occurs in the juxta-articular region [8]. Repetitive mechanical traumas have been suggested as a possible cause of tumoral calcinosis. Multiple micro-traumas cause transient hyperphosphatemia, leading to the accumulation and calcification of calcium-phosphate products [9]. Some reports suggest that degenerative spinal changes can contribute to spinal tumoral calcinosis [2,4,5]. Durant et al. [2] and Miura et al. [4] reported cases associated with vertebral compression fractures, concluding that spinal instability and resultant inflammation may contribute to the condition. Ebot et al. [5] described tumoral calcinosis in the cervical and lumbar spine due to degenerative changes. ASD is defined as any abnormal state that develops in a mobile segment adjacent to a spinal fusion, such as disc degeneration, listhesis, instability, hypertrophic facet joint arthritis, herniated nucleus pulposus, or stenosis [10]. Spinal fusion accelerates the progression of normal degenerative changes occurring at the adjacent level [11]. In our case, tumoral calcinosis developed adjacent to a previous lumbar spine fusion site, resulting in compression of the dura mater and subsequent neurological deficits. ASD after spine fusion may cause spinal instability, potentially resulting in the formation of spinal tumoral calcinosis.

Smack et al.'s [3] classification of tumoral calcinosis divides the disease into three subtypes: (1) primary normophosphatemic tumoral calcinosis occurs without metabolic abnormalities and shows no evidence of familial patterns; (2) primary hyperphosphatemic tumoral calcinosis has strong familial patterns; and (3) secondary tumoral calcinosis is associated with concurrent diseases that can cause soft tissue calcification, such as chronic renal failure, hyperparathyroidism, hypervitaminosis D, scleroderma, pseudoxanthoma elasticum, malignancy, and milk-alkali syndrome. Our patient had mild renal dysfunction but normal calcium and phosphate levels and no family history; therefore, we considered it a case of primary normophosphatemic tumoral calcinosis.

The treatment of choice for tumoral calcinosis of the spine, if it causes progressive neurological symptoms, is surgical removal of the lesion. Several reports indicate that total excision is an effective approach for patients with spinal tumoral calcinosis [12,13]. If total resection is impossible, clinical and radiographic follow-up are essential to identify any recurrence [8]. On the other hand, some favorable outcomes have been reported with laminectomy, partial lesion resection, and spinal fusion [5,14]. Guo et al. [14] reported a case where hemilaminectomy, fusion of the cervical spine, and removal of the calcified mass were performed, resulting in the disappearance of the remaining calcified mass two years after surgery. In our case, total excision was difficult due to the extensive anterior lesion and the risk of intraoperative neurological complications. However, neurological symptoms significantly improved after laminectomy to relieve spinal cord compression and extended fusion surgery, with no increase in calcified lesions observed postoperatively. This suggests the effectiveness of decompression and stabilization through surgical intervention in managing tumoral calcinosis caused by spinal instability.

## Conclusions

Tumoral calcinosis, although rare in the spine, should be considered in the differential diagnosis of spinal cord compression, especially following ASD after lumbar fusion. The pathogenesis may involve spinal instability. Even if total resection is not feasible, surgical intervention, including laminectomy with partial lesion resection for decompression and extended fusion for stabilization, may effectively improve neurological function and prevent further progression of the condition.
